# Robust perovskite formation via vacuum thermal annealing for indoor perovskite solar cells

**DOI:** 10.1038/s41598-023-37155-4

**Published:** 2023-07-06

**Authors:** Kwanchai Penpong, Chaowaphat Seriwatanachai, Atittaya Naikaew, Napan Phuphathanaphong, Ko Ko Shin Thant, Ladda Srathongsian, Thunrada Sukwiboon, Anuchytt Inna, Somboon Sahasithiwat, Pasit Pakawatpanurut, Duangmanee Wongratanaphisan, Pipat Ruankham, Pongsakorn Kanjanaboos

**Affiliations:** 1 School of Materials Science and Innovation, Faculty of Science, Mahidol University, Nakhon Pathom, 73170 Thailand; 2 Center of Excellence for Innovation in Chemistry (PERCH-CIC), Ministry of Higher Education, Science, Research and Innovation, Bangkok, 10400 Thailand; 3 National Metal and Materials Technology Center (MTEC), Khlong Luang, 12120 Pathum Thani Thailand; 4 Department of Chemistry, Faculty of Science, Mahidol University, Bangkok, 10400 Thailand; 5 Department of Physics and Materials Science, Faculty of Science, Chiang Mai University, Chiang Mai, 50200 Thailand

**Keywords:** Solar cells, Devices for energy harvesting

## Abstract

Perovskite materials are fascinating candidates for the next-generation solar devices. With long charge carrier lifetime, metal-halide perovskites are known to be good candidates for low-light harvesting. To match the irradiance spectra of indoor light, we configured a triple-cation perovskite material with appropriate content of bromide and chloride (FA_0.45_MA_0.49_Cs_0.06_Pb(I_0.62_Br_0.32_Cl_0.06_)_3_) to achieve an optimum band gap (E_g_) of $\sim$1.80 eV. With low photon flux at indoor condition, minimal recombination is highly desirable. To achieve such goal, we, for the first time, combined dual usage of antisolvent deposition and vacuum thermal annealing, namely VTA, to fabricate a high-quality perovskite film. VTA leads to compact, dense, and hard morphology while suppressing trap states at surfaces and grain boundaries, which are key culprits for exciton losses. With low-cost carbon electrode architecture, VTA devices exhibited average power conversion efficiency (PCE) of 27.7 ± 2.7% with peak PCE of 32.0% (Shockley–Queisser limit of 50–60%) and average open-circuit voltage (V_oc_) of 0.93 ± 0.02 V with peak V_oc_ of 0.96 V, significantly more than those of control and the vacuum treatment prior to heat.

## Introduction

Energy demand has been dramatically increasing owing to population explosion and industrial expansion. Metal-halide perovskite-based materials are fascinating candidates for the next-generation solar devices. Because of exceptional light to electricity conversion characteristic and rapid increase of PCE over time to more than 25%, they are extremely promising for future commercial applications with PCE rivaling those of silicon solar cells^1,2^. Furthermore, perovskite solar cells (PSCs) have good optoelectronic properties such as high absorption over the visible spectrum^3^, low exciton binding energies^4^, low non-radiative recombination rates^5^, long charge carrier diffusion lengths in the $\mu$m range, and tunable energy band gaps (E_g_) from 1.47 to more than 3.06 eV^6,7^. Interestingly, fabrication processes of PSCs are facile and economical with low consumption of energy due to high solubility at temperatures less than 100 °C^8,9^. Perovskite materials can be deposited by using several approaches, such as spin coating, vapor assisted solution deposition, thermal vapor deposition, inkjet printing, slot-die coating, and spray coating^10–13^. However, spin-coating deposition is a simple method for laboratory scale perovskite fabrication. One-step spin coating is a technique where precursor solution is directly spin–coated onto substrates^14,15^. Using antisolvent in one–step deposition, repeated cation doping, or adding some additives could be used to obtain large grain size, along with uniform and dense perovskite films^10,16^. Interestingly, E_g_ of perovskite-based materials with ABX_3_ structure can be tuned by substitution engineering at any site (A, B, and/or X), which is an advantage of perovskite technology, enabling several applications such as light emitting diodes (LEDs), photodetectors (PDs), and solar cells for both outdoor and indoor conditions. Up until now, researchers have paid a lot of attention to photovoltaics for low light that can be applied to portable electronics and wireless telecommunication technologies because of the rise of Internet of Things (IoTs)^17–19^. Perovskite–based materials are widely tunable in terms of energy band gaps, allowing a variety of E_g_ values each for an optimal performance under a specific light condition^20,21^. As indoor light sources provide different spectrum outputs compared to those of sunlight; according to computational calculation^21,22^, the well-fit energy band gap for indoor applications ranges from 1.8 to 1.9 eV, which can be achieved by partial substitution of A-site with cesium (Cs), methylammonium (MA), and/or formamidinium (FA) and/or X-site via iodine (I), bromine (Br), and/or chlorine (Cl)^22–26^. Cheng, R. et al. reported energy band gap tuning by the introduction of Br and Cl into pristine MAPbI_3_ perovskite. The E_g_ can be enlarged from 1.61 to 1.80 eV (MAPbI_2−x_BrCl_x_). Moreover, the addition of chlorine reduces trap-state density, halide migration, and non-radiative recombination while improving crystallization, resulting in high open circuit voltage (V_oc_) of 1.028 V and PCE of 36.2% under 1000 lux fluorescent light^21^. Cheng, R. et al. demonstrated that perovskite solar cell performance was improved, particularly for indoor light applications by chlorine additive. The chlorine doping contributes to higher extraction capability at the perovskite/hole transport layer interface, which is attributed to lower defects^19^. With proper addition of Br, the grain sizes of perovskite were enlarged, suppressing non-radiative recombination. Moreover, the stability was improved by the formation of a pseudo-cubic phase and PCE of 34.5% was realized^27^. Apart from the perovskite absorber layer, electron transport layer (ETL) also plays important roles. Ann, M. H. et al. reported that compact TiO_2_ (c-TiO_2_) layer was more efficient for indoor light applications than mesoporous TiO_2_ (m-TiO_2_) layer owing to the high density of interfacial traps from using m-TiO_2_, although m-TiO_2_ was better for one sun condition^28^. Dagar, J. et al. also reported tin oxide (SnO_2_) as the electron transport layer for perovskite solar cell tested under indoor illumination, showing PCE of 21.3% at 400 lux^29^. From previous studies, the trap states at the interfaces and/or the grain boundaries are the main sources of non-radiative recombination^22,23,28^. Especially for low light applications, trap-state density is of great importance as there are less photo-generated charges under indoor light environment. To circumvent this problem under one sun, researchers have paid attention to the application of vacuum treatment to boost nucleation during crystallization and remove residual solvents without using antisolvent^30–32^. Li, X. et al. applied vacuum treatment to fabricate FA_0.81_MA_0.15_PbI_2.51_Br_0.45_ in DMSO-GBL solvent system and put the wet film under a vacuum environment for a few seconds to promote DMSO-PbI_1.7_Br_0.3_-(FAI)_0.85_(MABr)_0.1_ intermediate phase by eliminating solvent. The intermediate phase could delay crystal growth, leading to larger grain size and a high PCE of 20.5% under one sun^30^. In another study, low band gap FA_0.8_MA_0.2_Sn_0.5_Pb_0.5_I_3_ was deposited by applying vacuum-assisted growth control (VAGC) instead of antisolvent technique; the wet spin-coated films were placed under vacuum at 10 Pa for 10 s and further annealed under N_2_ atmosphere, resulting in smooth surfaces, no pin holes, large columnar grain orientations, fast transportation of charge carriers, and improved charge-carrier lifetime^32^. Zhang, J. et al. fabricated quasi-2D PEA_2_MA_n-1_Pb_n_I_3n+1_ film by spin coating and vacuum treatment of the wet film to create uniform dispersion of different-n-value nanoplates to boost nucleations and limit the grain sizes by fast evaporation of residual solvents. The vacuum-treated films exhibited high fill factor (FF) of 82.4% and PCE of 18.04%^31^. Bi, D. et al. reported that perovskite solution (FA_0.9_Cs_0.1_PbI_3_) was added with molecular modulators (S, N, and SN) and fabricated by the one-step deposition method and then applied with a short vacuum treatment after spin coating to remove N, N-dimethylformamide (DMF) without using antisolvent to encourage fast crystallization of the intermediates. As a result, they achieved a PCE of over 20% with an active area of 1 cm^2^^33^. Vacuum treatment was also applied to two-step deposition during the perovskite formation when MAI was dropped onto a PbI_2_ film, resulting in rapid solvent removal and a supersaturated state where  a tremendous amount of nuclei are formed and simultaneously grown under the competitive pressure from neighbor nuclei; as a result, compact and smooth perovskite films with high hardness and thermal stability were produced^34^. Moreover, vacuum process can be applied during thermal annealing. Xie, F. X. et al. reported the CH_3_NH_3_PbI_3_ film fabricated by conducting vacuum treatment during thermal annealing to eliminate CH_3_NH_3_Cl (MACl), which is an unwanted byproduct of the reaction: 3CH_3_NH_3_I + PbCl_2_ $\to$ CH_3_NH_3_PbI_3_ + 2CH_3_NH_3_Cl, yielding a high PCE of 14.5%^35^. Feng, J. et al. reported Cs_0.15_FA_0.85_PbI_3_ films fabricated under an all vacuum process. The PbI_2_, FAI, and CsI were separately evaporated layer-by-layer; all precursor layers react to form complete perovskite by annealing under vacuum environment. As a result, they obtained PCE of 21.32%^36^.

**Figure 1:**
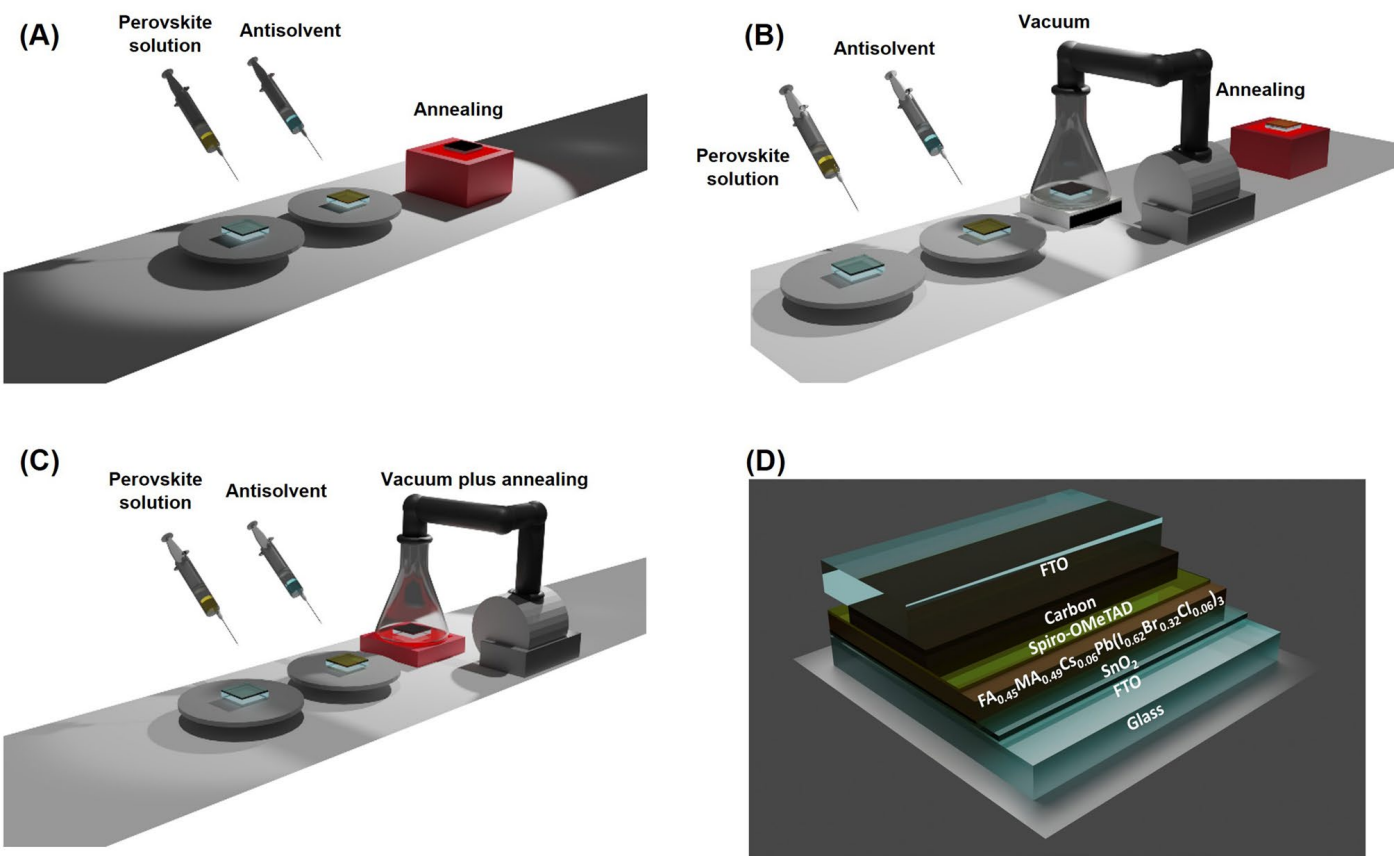
(**A**) Schematic of antisolvent and then thermal annealing (control), (**B**) antisolvent plus vacuum and then thermal annealing (VAC), (**C**) antisolvent plus vacuum thermal annealing (VTA), and (**D**) the graphical image of full device.

In this work, we aim to develop a novel process for low light perovskite materials. First, triple-cation perovskite with appropriate bromide and chloride was developed; the new perovskite recipe crafted the thin films with E_g_ of ~ 1.80 eV, close to the optimal E_g_ for indoor applications. We then combined both antisolvent deposition and vacuum thermal annealing, namely VTA, to produce a high quality perovskite layer. A triple-cation perovskite film was fabricated on a FTO/SnO_2_ substrate via one step deposition using chlorobenzene (CB) as an antisolvent. Then, we placed the sample inside a vacuum flask, which was connected to a vacuum pump with controllable pressure and located on top of a hotplate to regulate the temperature (Fig. 1). We achieved high performance triple-cation perovskite solar cells for low light application via the dual usage of antisolvent and vacuum thermal annealing. As indoor light intensity is at least 300 times lower than that of sunlight, dense and homogeneous perovskite formation enticed by vacuum thermal annealing is valuable.

## Methodology

Lead(II) iodide (PbI_2_; 99%) and lead(II) bromide (PbBr_2_; 98%) were purchased from TCI CO., LTD. Formamidinium bromide (FABr; ≥ 99%), methylammonium bromide (MABr; 98%), methylammonium chloride (MACl; 98%), cesium iodide (CsI; 99.9%, trace metals basis), anhydrous N,N-dimethylformamide (DMF; 99.8%v/v), anhydrous dimethyl sulfoxide (DMSO, 99%v/v), anhydrous chlorobenzene (ฺCB; 99.8%), anhydrous ethanol (ethanol; 99.5%v/v), tin(II) chloride dihydrate (SnCl_2_·2H_2_O; 99.999%), hydrochloric acid (HCl; 37%v/v), 4-tert-butylpyridine (tBP), lithium bis-(trifluoromethanesulfonyl) imide (Li-TFSI), anhydrous acetonitrile (acetonitrile; 99.8%), and 2,20,7,70-tetrakis[N,N-bis(4-methoxyphenyl)amino]-9,90-spirobifluorene (spiro-OMeTAD) were purchased from Sigma-Aldrich. Alconox detergent powder was purchased from Alconox. TEC 15 Fluorine-doped tin oxide (FTO) glass plates were purchased from Greatcell Solar. Carbon paste Jelcon CH-8 (low-resistance carbon paste with excellent performance) was from Jujo chemical.

2.5 × 2.5 cm^2^ FTO substrates were sonicated in an alconox solution, deionized (DI) water, and isopropanol, respectively. The substrates were then soaked in isopropanol for a night and blown with N_2_ gas. SnCl_2_·2H_2_O powder was dissolved in ethanol at 0.2 Molar and kept for 2 days at room temperature before use. Prior to ETL deposition, FTO substrates were treated in UV/ozone for 30 min for surface pretreatment. SnCl_2_ solution was filtered through a 0.22 m$\mu$ PTFE CNW syringe filter (ANPEL Laboratory Technology) and deposited onto 2.5 × 2.5 cm^2^ substrates via spin coating at 3000 rpm with initial acceleration of 1500 rpm/s for 30 s under ambient conditions. Then, the FTO/SnO_2_ substrates were annealed at 180 °C for 60 min and cooled down under room temperature. This method was used in our previous publication^37^.

Perovskite precursor solution was prepared at 1.3 M under N_2_ filled glovebox. The process is not simply a direct mixing of all precursors, as PbBr_2_, MACl, and CsI cannot be dissolved in DMF at high concentration. PbI_2_ (0.5786 g), PbBr_2_ (0.0384 g), and MACl (0.0202 g) were weighed in the same vial. FABr (0.1875 g) and MABr (0.1680 g) were separately dissolved in 1000 μL of 6:1 DMF:DMSO solvent mixture and CsI (0.0780 g) was dissolved in 200 $\mu$L of DMSO as stock solutions; all vials were separated and stirred for 30 min at room temperature. Then, 466 $\mu$L of FABr solution, 314 $\mu$L of MABr solution, 66 $\mu$L of CsI solution, and 200 $\mu$L of 6:1 DMF:DMSO solvent were added into the mixed powder vial. Perovskite solution was stirred for 8 h at 70 °C and filtered through a 0.22 $\mu$m PTFE CNW syringe filter. Before deposition, FTO/SnO_2_ substrates were cleaned in a UV ozone cleaner. All perovskite films were spin coated under N_2_ environment in a glovebox. The 1.3 M perovskite solution was deposited by spin coating at 1500 rpm with initial acceleration of 750 rpm/s for 100 s onto substrates and chlorobenzene was then dropped at 21 s as the antisolvent after starting the spin program. Finally, the samples were annealed at 100 °C for 30 min under N_2_ environment in a glovebox as control samples. For the antisolvent plus vacuum and then thermal annealing process (VAC), the perovskite films were first prepared in the same method as that of control; however, the samples were placed in vacuum treatment at 80 Pa for 30 min prior to annealing at 100 °C for 30 min in N_2_ environment. With VTA samples, the perovskite films were also prepared in the same way as that of VAC but the annealing at 100 °C for 30 min was conducted at the same time with the vacuum treatment at 80 Pa for 30 min by placing a hotplate under the vacuum flask. All experimental conditions were summarized in Fig. 1A–C.

The hole transport material (HTM) was prepared by dissolving 80 mg of spiro-OMeTAD in 1 mL of chlorobenzene and 520 mg of Li-TFSI in 1 mL of acetonitrile. Both solutions were stirred for at least 2 h before use. Then, 28.5 µL of 4-tert-butylpyridine and 17.5 µL of Li-TFSI solution were added into the spiro-OMeTAD solution and stirred at room temperature for 24 h. The solution with volume of 60 $\mu$L was dropped and left for 30 s on top of the perovskite layer before starting the spin-coating process with spin speed of 2000 rpm for 30 s at 1000 rpm/s acceleration. The deposited samples were kept in a glovebox for 1 night.

80 $\mu$m of carbon layer was used as the top electrode, which was prepared by the doctor blading method. Clean glass substrate was taped with four layers of Kapton tape on two sides, leaving a well in between. Carbon paste was then applied onto the well. The wet carbon film was soaked in ethanol for 2 h. After 2 h, the carbon sheet was removed from the glass substrate and dried at room temperature for 30 min to eliminate residual ethanol. The carbon sheet was pressed by a pressing machine at 0.6 MPa at room temperature for 5 min and could be cut for further usage. Approximately 0.04 cm^2^ of square carbon sheet was placed on top of HTM and then covered by FTO glass. This method was modified from previous publication^38^. Eventually, the sandwich-structure device was pressed at 0.6 MPa at 70 °C for 5 min to finish the full device. The graphical image of full device was shown in Fig. 1D.

The crystal structure was characterized by Bruker, D8 Discover X-ray diffractometer (Cu anode material, detector scan mode using a step size of 0.02°, 0.6 s per step, and 2θ from 5° to 50°). Surface morphologies and cross-sections were observed by scanning electron microscopy (FE-SEM; JSM-7610FPlus JEOL, 20 kV, and secondary electron mode). The optical absorption spectra were obtained by using a Shimadzu UV-2600 UV–Vis spectrophotometer (800–400 nm, medium mode, and absorbance mode). The photoluminescence spectra were recorded by Horiba FluoroMax+ spectrofluorometer (integration time of 0.1 s, excitation of 560 nm, excitation slit of 8 nm, emission wavelength measurement between 550 and 850 nm, and emission slit of 8 nm). Photoluminescence lifetime (PL-lifetime) of the samples was measured by Horiba FluoroMax+ . A nanoLED diode emitting 625 nm pulses at 1 MHz was used as an excitation source with bandpass of 20 nm and sync delay of 40 ns. EQE, responsivity, and specific detectivity were measured using Enlitech QE-R quantum efficiency analyzer (DC mode with 0.04 mm^2^ beam diameter). The topography, short-circuit current mapping, and open-circuit voltage mapping were performed by conductive atomic force microscope (C-AFM) via Park NX10 using a conductive probe (ANSCMPC, k = 0.036 N/m, and resonance frequency = 15 kHz). The measurement setup was done with a contact force setpoint of 1.8 nN and a scan speed of 2.5 μm/s. The sample biases are set to 0 V for the short circuit current mapping and 0.6 V (forward bias) for the open circuit voltage mapping while being excited with the microscope's white light with the power 0.2 mW/cm^2^ in ambient air (~70% RH) at room temperature (~25 °C).

One-sun illumination (100 mW/cm^2^) was provided by 7520-LED light source with LSS-7120 LED controller (VeraSol). 4 W LED 6500 K (Philipe, E27, cool daylight) was used as indoor light source. The light intensity was calibrated by Si diode (Hamamatsu S1133). The active area of each cell is 0.04 cm^2^. The photocurrent density–voltage (J-V) curves were measured from 1.00 V to −0.10 V under indoor light at 1000 lux (0.31 mW/cm^2^) and 1.10 V to −0.10 V under one sun with a scan step of 0.02 V and a delay time of 500 ms. The measurement was done under ambient air at room temperature without any encapsulation. The device stability was measured every two days for devices with encapsulation under ambient air at room temperture and under both 1000 lux and one sun; the devices were kept under 1000 lux for 8 h/day.

## Results and discussion

**Figure 2:**
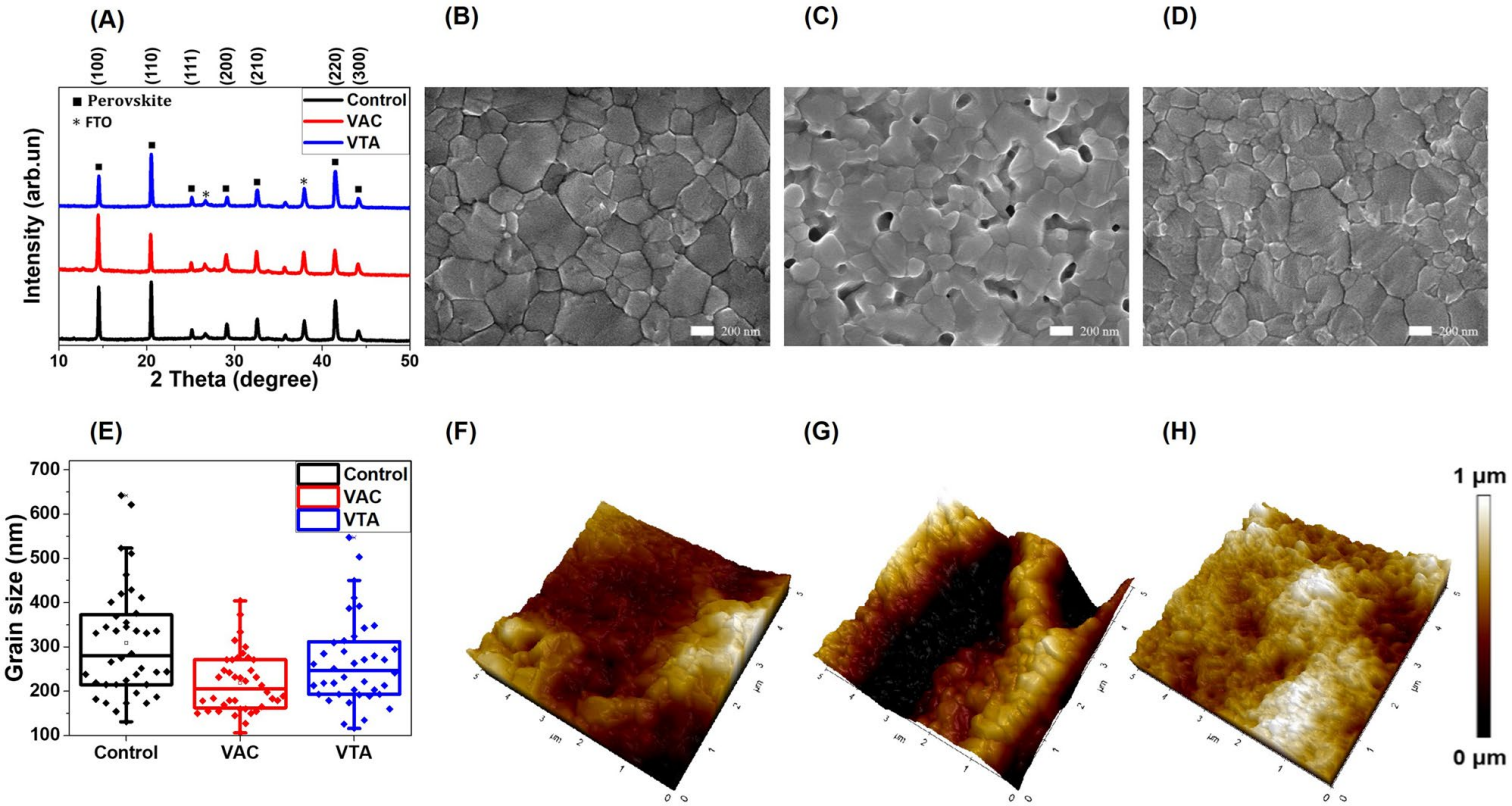
(**A**) XRD patterns of control, VAC, and VTA samples. (**B**–**D**) SEM top surface images from using control, VAC, and VTA samples. (**E**) The average grain sizes of control, VAC, and VTA samples. (**F**–**H**) AFM 3D-images of control, VAC, and VTA samples.

The XRD patterns of perovskite films fabricated with and without VTA are illustrated in Fig. 2A. We found full transformation into perovskite thin films since PbI_2_ and hexagonal non-perovskite phase (δ-phase) peak could not be detected^36,39^. The XRD peak of our samples located at 14.5° corresponds to the crystallographic plane of (100), which is cubic phase^40^. The peak positions of perovskite are shifted to higher 2$\uptheta$ degrees compared with our reference, which is around 14.0°^40^. According to Bragg’s law,

1 $$2\text{d} \, \sin \theta = \, \text{n}\lambda ,$$

**Figure 3:**
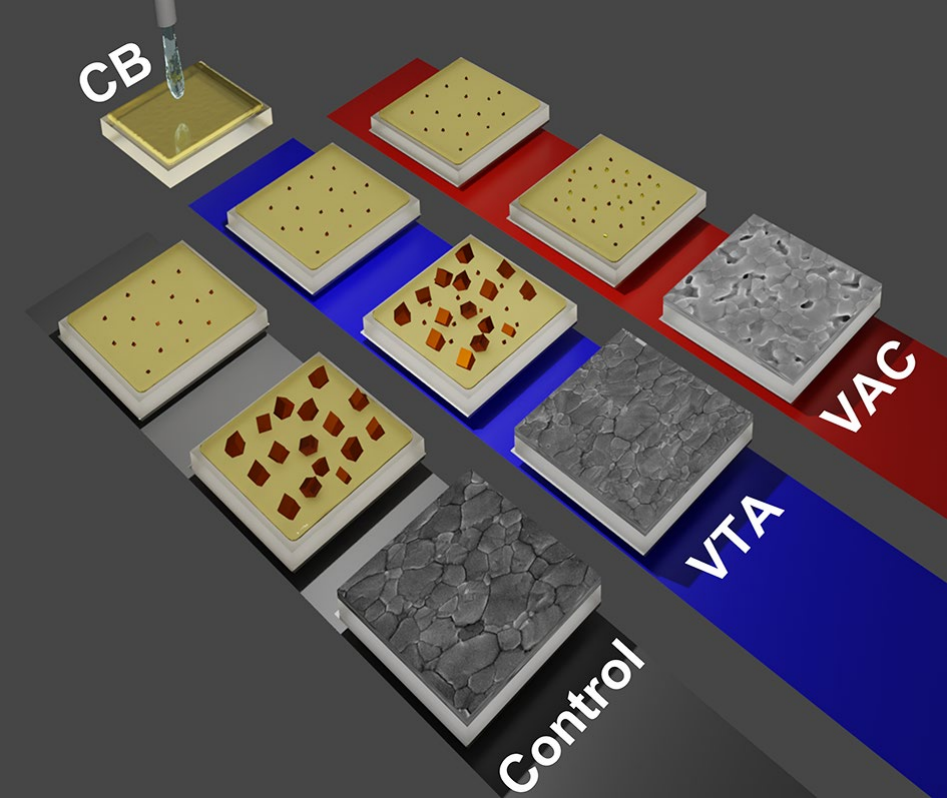
Proposed mechanism of control, VAC, and VTA processes.

The increase in $\uptheta$ indicates a decrease in d-spacing (d), accompanying partial substitution of larger I with smaller Br and/or Cl. Furthermore, according to the full width at half maximum (FWHM), the crystallite sizes were calculated by using the Scherrer equation at the (100) peaks. The average crystallite sizes are 48.1, 41.7, and 46.9 nm for control, VAC, and VTA, respectively. The crystallite sizes of all conditions are quite similar. However, the average crystallite size of control is the largest and that of VAC is the smallest. The average crystallite size of VTA is between those of control and VAC. The morphology of perovskite surfaces are examined by SEM (Fig. 2B–D) and atomic force microscopy (AFM) (Fig. 2F–H). Interestingly, we find that VTA perovskite formation is the most compact with the smallest roughness among the three with an average grain size of 265.3 ± 97.3 nm. While VAC results in the smallest average grain sizes of 218.5 ± 67.1 nm with many pinholes and high roughness. In contrast, the control condition has the biggest grain with an average grain size of 300.5 ± 121.7 nm along with medium roughness. VTA strikes the right balance, achieving good grain size and low roughness. SEM cross section images of all conditions are exhibited in Fig. S1. The control shows clear vertical grain boundaries, while VTA has the same trend with control. In contrast, the perovskite film processed with VAC shows randomly stacked small-size grains with pinholes. Vertical grains are beneficial, causing less recombination as the photogenerated carriers can pass through the active layer and reach the corresponding charge carrier extraction interfaces. VAC and VTA films are thinner when compared to the control film using the same concentration of perovskite solution, suggesting dense formation with vacuum. We hereby propose a mechanism based on a modified LaMer model^16^. In general, the crystal formation is divided into three phases. In phase I, the precursor concentration of the solution increases and reaches supersaturation (C*). In phase II, nuclei are formed when the precursor concentration exceeds C*. In phase III, nucleation stops and crystal growth pursues when concentration is between the saturation concentration (C_s_) and C*. For the control sample, the triple-cation perovskite precursor solution is first spin-coated on top of ETL and antisolvent is dropped onto the rotating substrate to evaporate solvent, resulting in C* and nuclei generation. The concentration moderately reaches the C^*^ region, resulting in a number of nuclei as primary nuclei. With thermal annealing after spin coating, the nucleation is stopped as the C* and C_s_ levels rise with temperature. While concentration is between C* and C_s_ (phase III), heat additionally helps remove DMSO from the intermediate phase^30^, resulting in crystal growth with large grain sizes as seen in many reports^6,41–44^. The film becomes shiny brown during the annealing process. For VTA sample, antisolvent is used to induce some amounts of nuclei called as primary nuclei similar to that of control. After antisolvent, the sample was thermally annealed in a vacuum chamber, which initiates secondary nuclei by fast evaporation at the surface^30–32^. In this step, we hypothesize that there are two concurring events; primary nuclei grow vertically and horizontally due to heat similar to that of control while C* at the surface creates secondary nuclei, which then grow due to heat to fill up the gaps between crystals of the primary nuclei. Smaller grains can be seen between larger grains as shown in Fig. 2D, resulting in smooth and compact morphology. The film becomes shiny brown in this vacuum annealing process. For antisolvent plus vacuum condition, antisolvent induces some amounts of nuclei as discussed previously. After antisolvent, the sample is moved to a vacuum chamber without heat. In this step, C* at the surface mostly creates secondary nuclei while reducing the amount of solvent without crystal growth as, without heat, concentration remains at C*. The film becomes hazy without brown color in this process. In the last step, heat is applied for 30 min; with removal of DMSO in the intermediate state, both primary and secondary nuclei grow at the same time with limited remaining solvent and therefore less degree of freedom for crystal formation. As a result, the VAC film shows much smaller grain without interconnection, as there is not enough activation energy to induce the desired transition process and facilitate compact grain growth^44,45^, leading to rough morphology as seen in Fig. 2C. The film becomes hazy with brown color in the annealing process. The average grain size of control, VAC, and VTA samples were shown in Fig. 2E. The graphically proposed mechanism was exhibited in Fig. 3 and the modified LaMer model of control, VAC, and VTA was shown in Fig. S2. With hardness measurement as shown in Table S1, the vacuum processes in both VTA and VAC result in mechanically harder films as compared to the control in agreement with previous publication^34^.

**Figure 4:**
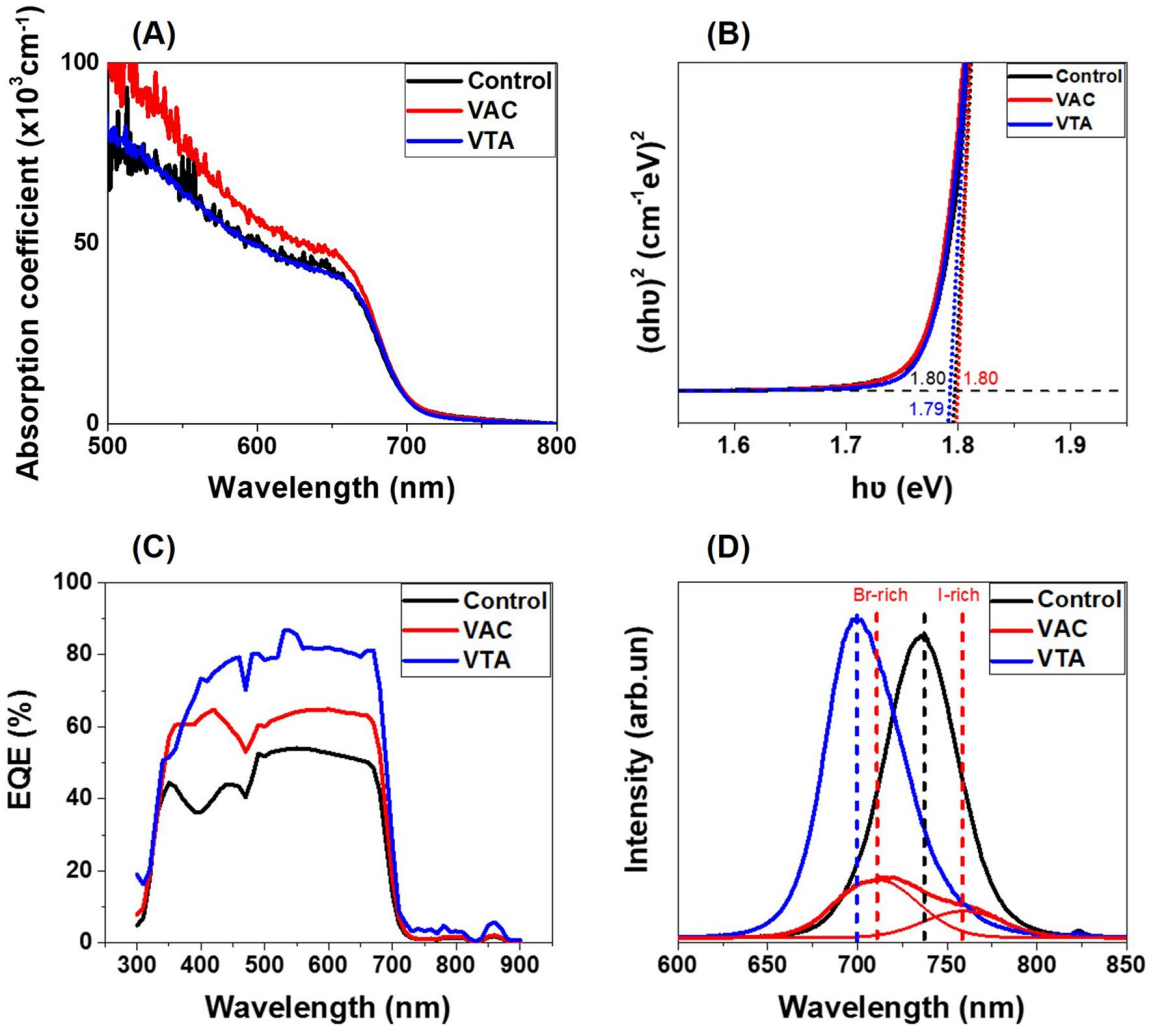
(**A**) The absorption coefficient for control, VAC, and VTA films. (**B**) Tauc plot for optical band gap (E_g_) analysis. (**C**) External quantum efficiency (EQE) spectra for control, VAC, and VTA devices. (**D**) Steady-state PL spectra of control, VAC, and VTA films.

The absorption coefficients and the Tauc plots of perovskite films are shown in Fig. 4A and 4B. Because of different thicknesses among control, VAC, and VTA samples, the absorption coefficients^46^ were calculated for comparison. The VAC sample shows highest absorption coefficient as the film is rough and scatters more light^47^. Using the suitable amount of I, Br, and Cl, the absorption edges of all conditions are ~ 712 nm, exhibiting the band gap close to 1.80 eV, located at the lower range of optimal values for low light applications (1.80 − 1.90 eV). The similar values are expected since the physical changes due to vacuum should not affect chemical composition. These results are consistent with external quantum efficiency (EQE) as shown in Fig. 4C. The EQE measurement is used to measure the ratio of generated electrons to given photons at a specific wavelength of light excitation. The generated electrons only occur in the light absorption range as expected.

The steady-state photoluminescence (PL) spectra are shown in Fig. 4D to evaluate the charge transfer dynamics of perovskite films. The perovskite films were fabricated on FTO/SnO_2_ substrates. For VTA, the PL intensity is the strongest, indicating lower non-radiative recombinations^36^ because of the most compact film and less grain boundaries. In the PL spectra, VAC film shows the lowest PL intensity possibly due to high grain boundaries and pin holes, which contribute to non-radiative recombination. The PL peak of the VAC case shows sign of phase separation with Br-rich peak near 720 nm and I-rich peak near 770 nm. The VTA peak position is blue-shifted compared to that of control, signaling decreased spontaneous radiative recombination caused by trap states on the surface and/or grain boundaries^48,49^.

To further investigate about the trap states, time-resolved photoluminescence was conducted on perovskite films. The photoluminescence lifetime (PL-lifetime) spectra of the perovskite on cleaned glasses were fitted bi-exponentially, with a slow decay component (${\varvec{\tau}}$_1_) and a fast decay component (${\varvec{\tau}}$_2_)^50^ as shown in Fig. S3. ${\varvec{\tau}}$_1_ and ${\varvec{\tau}}$_2_ are linked with charge recombination in the bulk and at the interfaces^36^, respectively. As shown in Table S2, the VTA sample has the longest ${\varvec{\tau}}$_1_, ${\varvec{\tau}}$_2_ and average decays compared to those of control and VAC samples, indicating lower electronic traps in the bulk and at the surface in agreement with the PL spectra as previously discussed. However, for PL-lifetime, perovskite films were formed on glass instead of FTO/SnO_2_ to reduce quenching and might lead to different adhesions and film morphologies. Therefore, we did further investigation to identify perovskite electronic traps.

**Figure 5:**
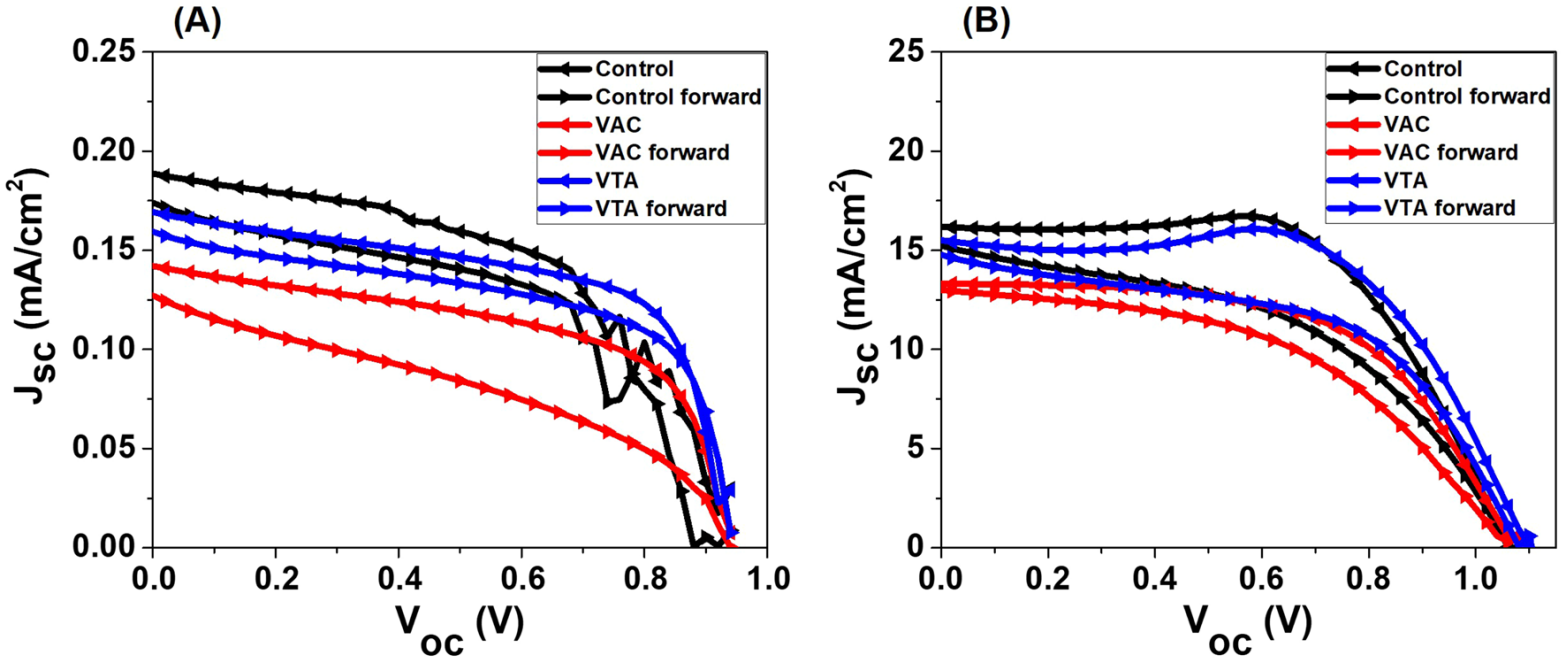
(**A**) The J-V curves of the best PSC devices under indoor light and (**B**) one sun.

**Figure 6:**
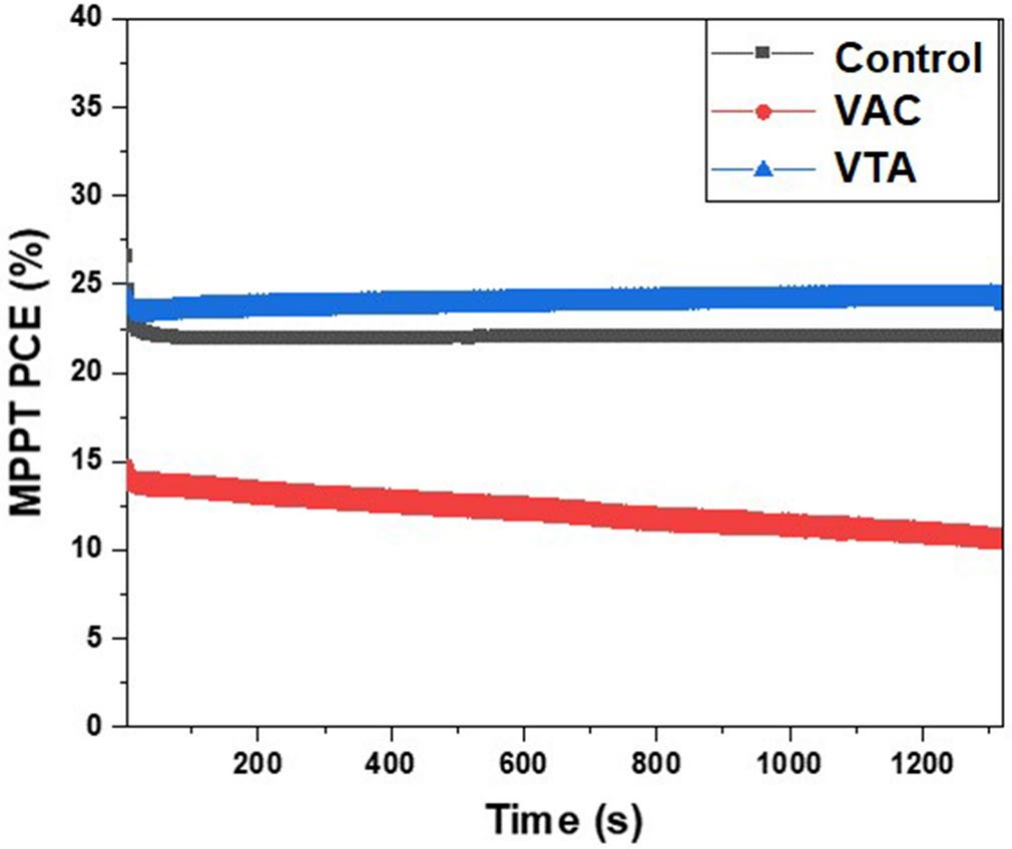
The maximum power point tracking (MPPT) of the PSC devices under indoor light.

We used two sources of light spectra which were one sun irradiation (AM1.5G, light intensity I_L_: 100 mW/cm^2^) and LED (illuminance: 1000 lux, light intensity I_L_: 0.31 mW/cm^2^) to investigate the incident light-dependent photovoltaic performances. The average photovoltaic parameters, which include V_oc_, J_sc_, FF, and PCE are summarized in Table 1 and the J-V curves of the best PSC devices are displayed in Fig. 5. The device parameters and hysteresis indexes (HI) from the J-V curves in Fig. 5 are shown in Table 2. Under 1000 lux, the control devices show PCE of 25.5 ± 3.0%, V_oc_ of 0.91 ± 0.02 V, and J_sc_ of 0.16 ± 0.02 mA/cm^2^ with peak PCEs of 30.7 and 27.0% from reverse and forward scans, respectively. However, for VAC, the solar performance drops with PCE of 19.9 ± 2.9%, V_oc_ of 0.90 ± 0.03 V, and J_sc_ of  0.12 ± 0.01 mA/cm^2^ with peak PCEs of 24.5 and 14.6% from reverse and forward scans, respectively. Interestingly, these values are enhanced for VTA, having PCE of 27.7 ± 2.7%, V_oc_ of 0.93 ± 0.02 V, and J_sc_ of  0.16 ± 0.01 mA/cm^2^ with peak PCEs of 32.0 and 28.3% from reverse and forward scans, respectively. Shockley–Queisser limit with E_g_ of 1.8–1.9 eV under low-light LED sources is ranged from 50 to 60%^22^. Higher V_oc_ of VTA corroborates less defect density due to good grain sizes and smooth surface in agreement with the morphological investigations as previously discussed. J_sc_ is lowest for VAC possibly due to bad contact from many pinholes as seen in SEM and AFM results. Fig. S4 and S5 shows the statistics of PCE, V_oc_, J_sc_, FF, R_shunt_ (R_sh_), and R_series_ (R_s_) under indoor light and one sun light sources. All of the device parameters under low light and one sun are listed in Table S3 and S4, respectively. Maximum power point tracking (MPPT) is shown in Fig. 6 to assess the correct power conversion efficiency; VTA results in superior PCE values compared to those of control and the VAC under indoor light. These results are consistent with the average PCEs in Table 1. The MPPT graphs of control and VTA are stable during measurement. However, the MPPT graph of VAC continuously decreases during the measurement. Moreover, long-term device stability and film-only stability for control, VAC, and VTA samples were shown in Figs. S6, S7, and S8.

**Table 1:** Averages photovoltaic parameters of control, VAC, and VTA devices under low light illumination (1000 lux).

Light source	Condition	V_oc_ (V)	J_sc_ (mA/cm^2^)	FF	PCE (%)
1000 lux	Control	0.91 ± 0.02	0.16 ± 0.02	0.50 ± 0.05	25.5 ± 3.0
AM1.5G		1.05 ± 0.03	12.2 ± 1.7	0.49 ± 0.08	7.2 ± 1.9
1000 lux	VAC	0.90 ± 0.03	0.12 ± 0.01	0.45 ± 0.07	19.9 ± 2.9
AM1.5G		0.96 ± 0.21	9.5 ± 4.3	0.40 ± 0.11	5.4 ± 3.5
1000 lux	VTA	0.93 ± 0.02	0.16 ± 0.01	0.48 ± 0.09	27.7 ± 2.7
AM1.5G		1.05 ± 0.05	13.6 ± 3.7	0.51 ± 0.06	8.6 ± 2.4

**Table 2:** Device parameters of the J-V curves in Fig. 5.

Sample	Scan direction	J_sc_ (mA/cm^2^)	V_oc_ (V)	FF	PCE (%)	HI
Low light						
Control	Reverse	0.19	0.93	0.54	30.7	12
	Forward	0.17	0.88	0.55	27.0	
VAC	Reverse	0.14	0.92	0.57	24.5	40
	Forward	0.13	0.93	0.38	14.6	
VTA	Reverse	0.17	0.93	0.63	32.0	12
	Forward	0.16	0.94	0.59	28.3	
One sun						
Control	Reverse	16.2	1.06	0.63	10.8	30
	Forward	15.2	1.06	0.47	7.6	
VAC	Reverse	13.3	1.07	0.58	8.2	20
	Forward	13.0	1.05	0.49	6.6	
VTA	Reverse	15.5	1.10	0.64	10.8	21
	Forward	14.8	1.08	0.53	8.5

**Figure 7:**
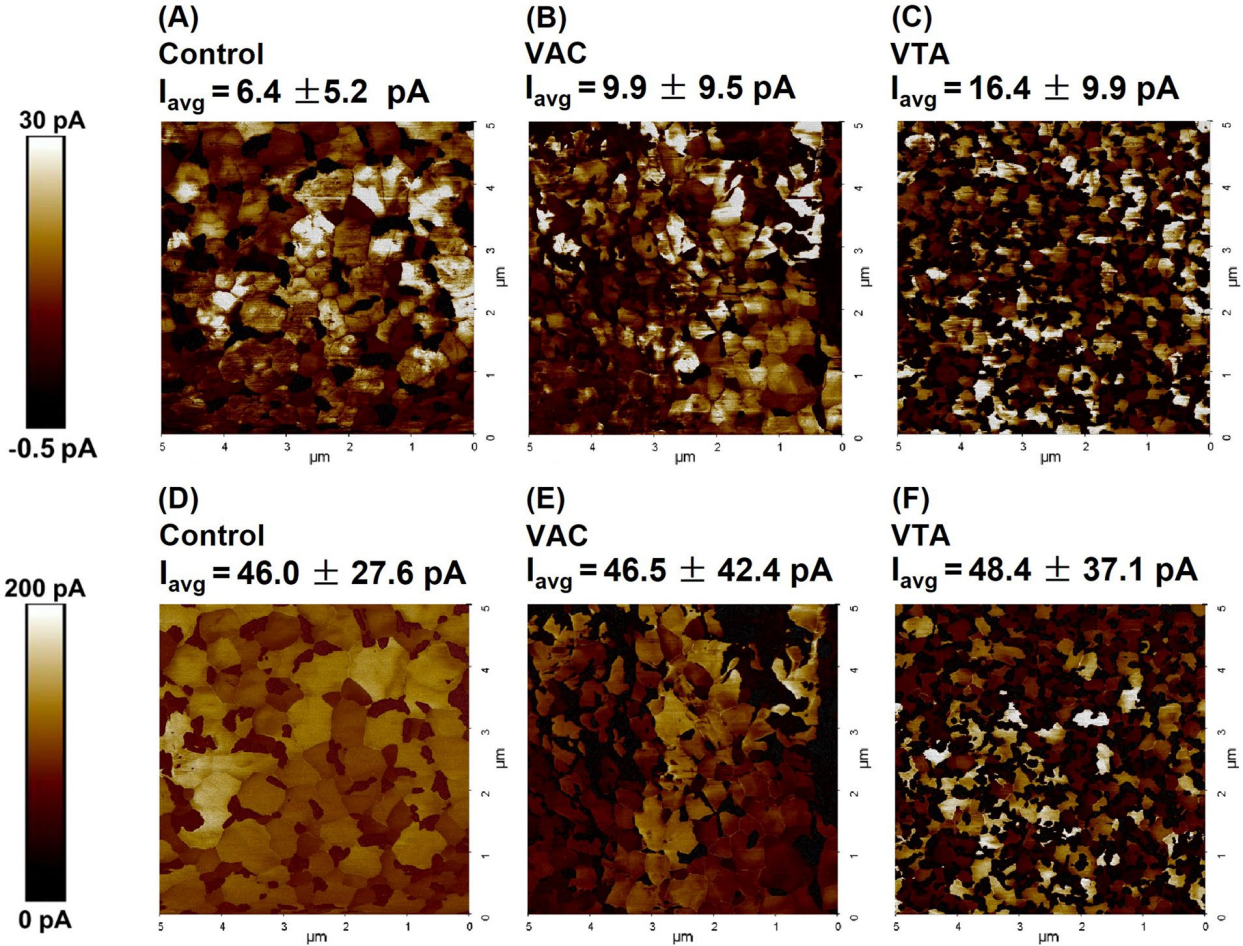
(**A**–**C**) The open-circuit voltage (V_oc_) mapping for control, VAC, and VTA. (**D**–**F**) The short-circuit current density (I_sc_) mapping for control, VAC, and VTA.

To investigate current-morphology correlation at the nanoscale, C-AFM was conducted for control, VAC, and VTA devices under 0.2 mW/cm^2^ illumination. The open-circuit voltage map (V_oc_ map) is done to simulate the V_oc_ condition in the actual solar cell device where voltage is maximized while current is close to zero. As the C-AFM study is performed solely on the FTO/SnO_2_/perovskite stack, the average V_oc_ is assumed to be 0.6 V instead around 1.0 V in the actual device as shown in Table 1 to account for imperfections due to the lack of the hole transport layer (HTL). In this mode, the cantilever tip is engaged with numerous nanoscale areas pixel by pixel while applying a forward bias of 0.6 V. In this V_oc_ map, the area with negative current represents the region where V_oc_ is less than 0.6 V and vice versa. As V_oc_ is linked to trap density, the negative region denotes perovskite surface with higher trap density^51^. As shown in Fig. 7A–C, the VTA sample shows the homogeneous current distribution over the measured area and the highest average positive current (I_avg_) of 16.4 ± 9.9 pA, larger in comparison to 9.9 ± 9.5 pA and 6.4 ± 5.2 pA from those of VAC and control, respectively, indicating much higher V_oc_ value on average and overall less traps for VTA. This superior display of I_avg_ from the VTA sample can be caused by reduction of surface trap state, contributing to lower recombination compared to those of control and VAC^51^. Interestingly, the regions with high currents of the V_oc_ map coincide with perovskite cores, indicating lower traps on the perovskite surface not at the boundaries^51^. These results are consistent with blue shifting in photoluminescence spectra and longer fast decay (${\varvec{\tau}}$_2_) from PL lifetime, which are linked to less surface/ boundary recombinations. Higher deviation of I_avg_ in Fig. 7B for VAC compared to that of control is correlated with the wider distribution of the V_oc_ as shown in photovoltaic measurements in Fig. S4. However, the higher I_avg_ from VAC samples over control, however, does not necessarily point toward less traps in actual solar devices as VAC samples additionally have large roughness, which, in turn, could further induce interfacial traps between perovskite and HTL not evaluated by the V_oc_ surface study.

Furthermore, the short circuit current map (I_sc_ map) was done to evaluate charge conductivity pixel by pixel at zero bias under 0.2 mW/cm^2^ illumination. Figure 7D–F show the I_sc_ map on the surfaces. The VTA sample has the highest I_avg_ of 48.4 ± 37.1 pA due to robust formation as compared to 46.5 ± 42.4 pA and 46.0 ± 27.6 pA from VAC and control, respectively. Naturally, the trend of average currents from I_sc_ maps is in line with J_sc_ results from Table 1. The I_sc_ maps clearly illustrate high conductivity at perovskite cores for VTA sample, indicating superior charge transport^52^ not at the boundaries as observed in the V_oc_ map. The poor J_sc_ results in VAC devices as compared to the relatively good I_avg_ from c-AFM could possibly be explained by large morphological distribution, as observed from rough SEM surface in Fig. 2C along with high roughness in Fig. 2G.

## Conclusions

In this work, we utilized dual usage of antisolvent deposition and vacuum thermal annealing to create a new perovskite treatment method, namely VTA, which is capable of fabricating a high-quality perovskite film. This approach leads to robust perovskite formation, as secondary nuclei induced by the vacuum treatment simultaneously grow to fill the gaps left by perovskite crystals stemmed from primary nuclei. As a result, VTA enables thin films to be denser and harder as compared to those of the traditional antisolvent method. Moreover, VTA leads to reduced surface roughness and electronic traps both at the bulk and the surface. With the VTA process, we obtained the average PCE of 27.7 ± 2.7% (peak PCE of 32.0%) along with V_oc_ of 0.93 ± 0.02 V (peak V_oc_ of 0.96 V) under 1000 lux illumination by using low-cost carbon as the electrode. The photovoltaic performances are significantly better than those of control and VAC. VTA opens doors for robust perovskite formation and could practically be adapted for various perovskite compositions towards desirable optoelectronic applications for modern society.

## Supplementary Information

Supplementary Information.

## Data Availability

The datasets used and/or analysed during the current study available from the corresponding author on reasonable request.
