# Supplementary material for: Robust perovskite formation via vacuum thermal annealing for indoor perovskite solar cells
Source: Sci Rep. 2023 Jul 6;13:10933. doi: 10.1038/s41598-023-37155-4 (PMC10325999; doi:10.1038/s41598-023-37155-4)
Supplement: Supplementary file 1 — Supplementary Information. [file 41598_2023_37155_MOESM1_ESM.docx]

**Robust perovskite formation via vacuum thermal annealing for indoor perovskite solar cells**

Kwanchai Penpong^1,2^, Chaowaphat Seriwatanachai^1^, Atittaya Naikaew^1^, Napan Phuphathanaphong^1^, Ko Ko Shin Thant^1^, Ladda Srathongsian^1^, Thunrada Sukwiboon^1^, Anuchytt Inna^1^, Somboon Sahasithiwat^3^, Pasit Pakawatpanurut^2,4^, Duangmanee Wongratanaphisan^5^, Pipat Ruankham^5^, Pongsakorn Kanjanaboos^1,2^*

^1^School of Materials Science and Innovation, Faculty of Science, Mahidol University, Nakhon Pathom 73170, Thailand

^2^Center of Excellence for Innovation in Chemistry (PERCH-CIC), Ministry of Higher Education, Science, Research and Innovation, Bangkok 10400, Thailand

^3^National Metal and Materials Technology Center (MTEC), Khlong Luang, Pathum Thani 12120, Thailand

^4^Department of Chemistry, Faculty of Science, Mahidol University, Bangkok 10400, Thailand

^5^Department of Physics and Materials Science, Faculty of Science, Chiang Mai University, Chiang Mai 50200, Thailand

[*pongsakorn.kan@mahidol.edu](mailto:*pongsakorn.kan@mahidol.edu)

**Supplementary materials**


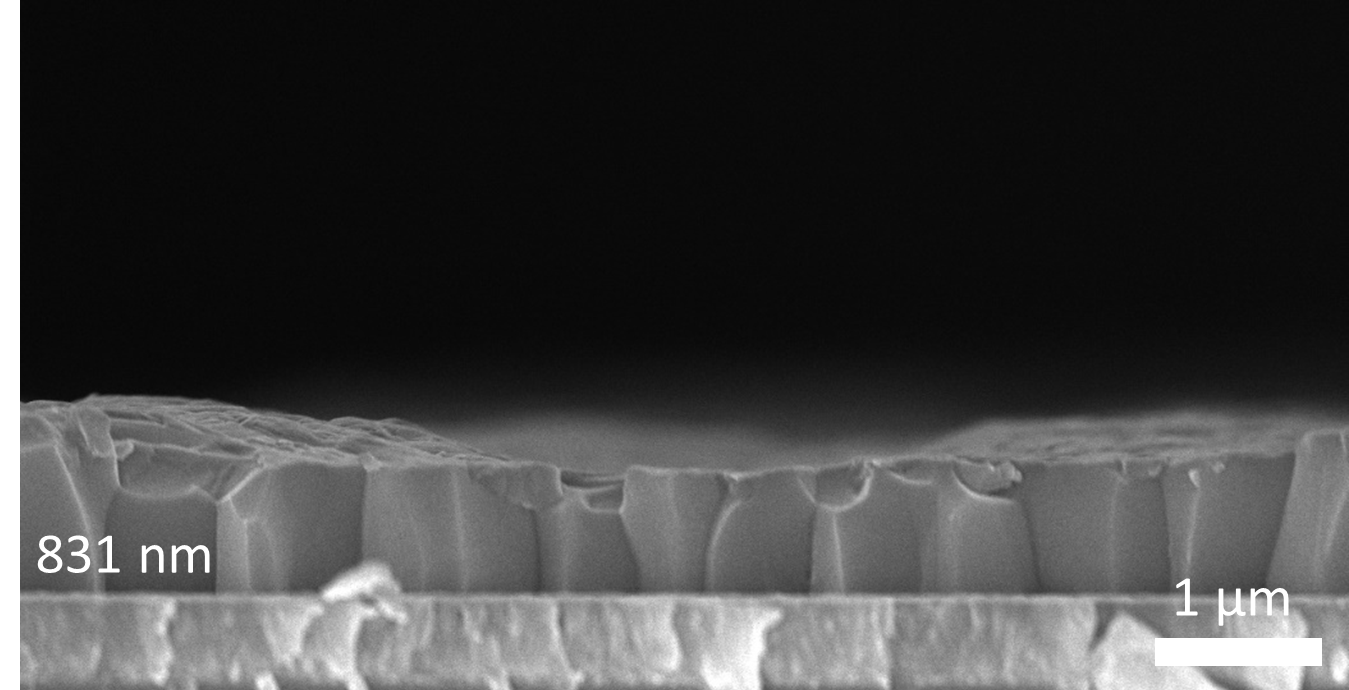


**Control**

**(A)**


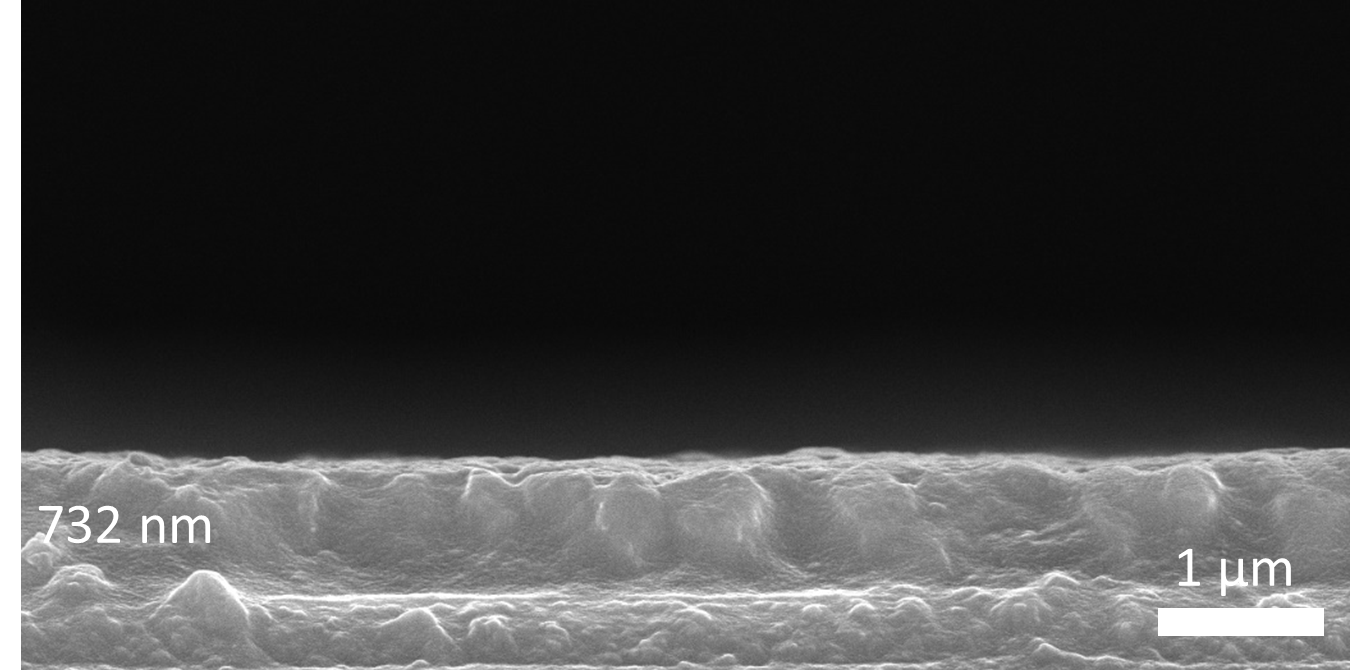


**VAC**

**(B)**


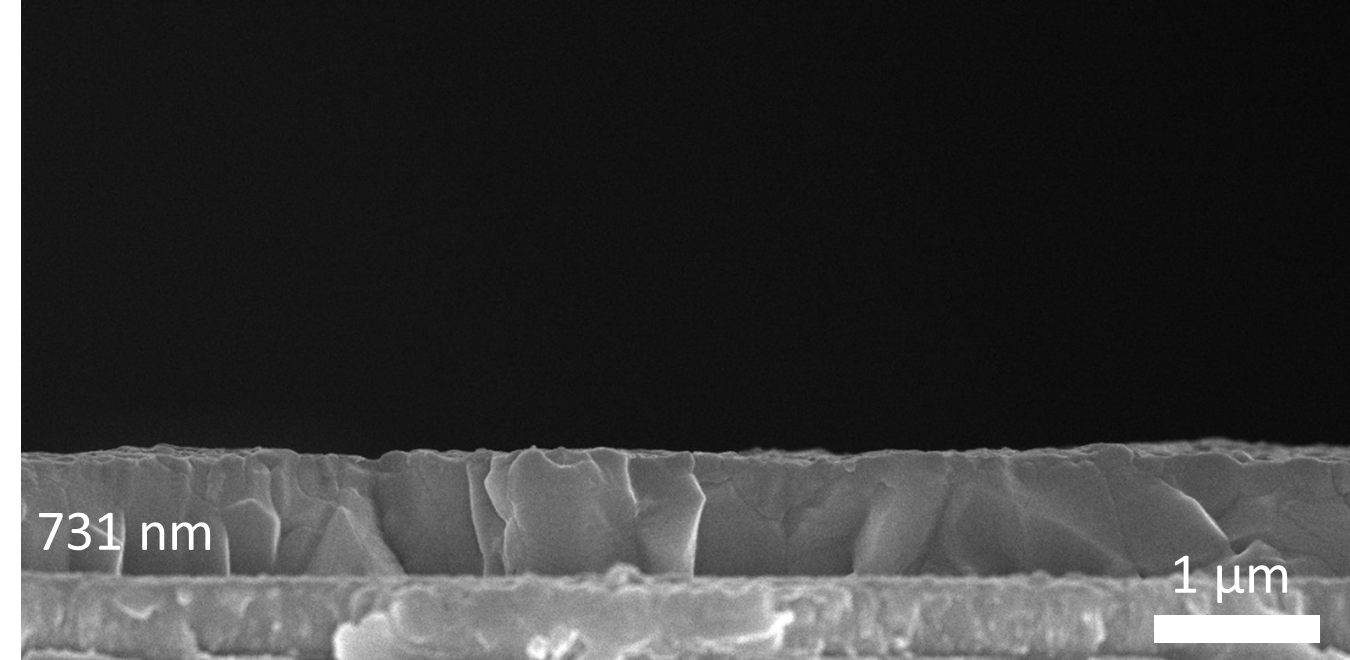


**VTA**

**(C)**

**Figure S1**. (A)-(C) SEM cross section images for control, VAC, and VTA films.

**Table S1.** Mechanical properties [1] of control, VAC, and VTA thin films.

| 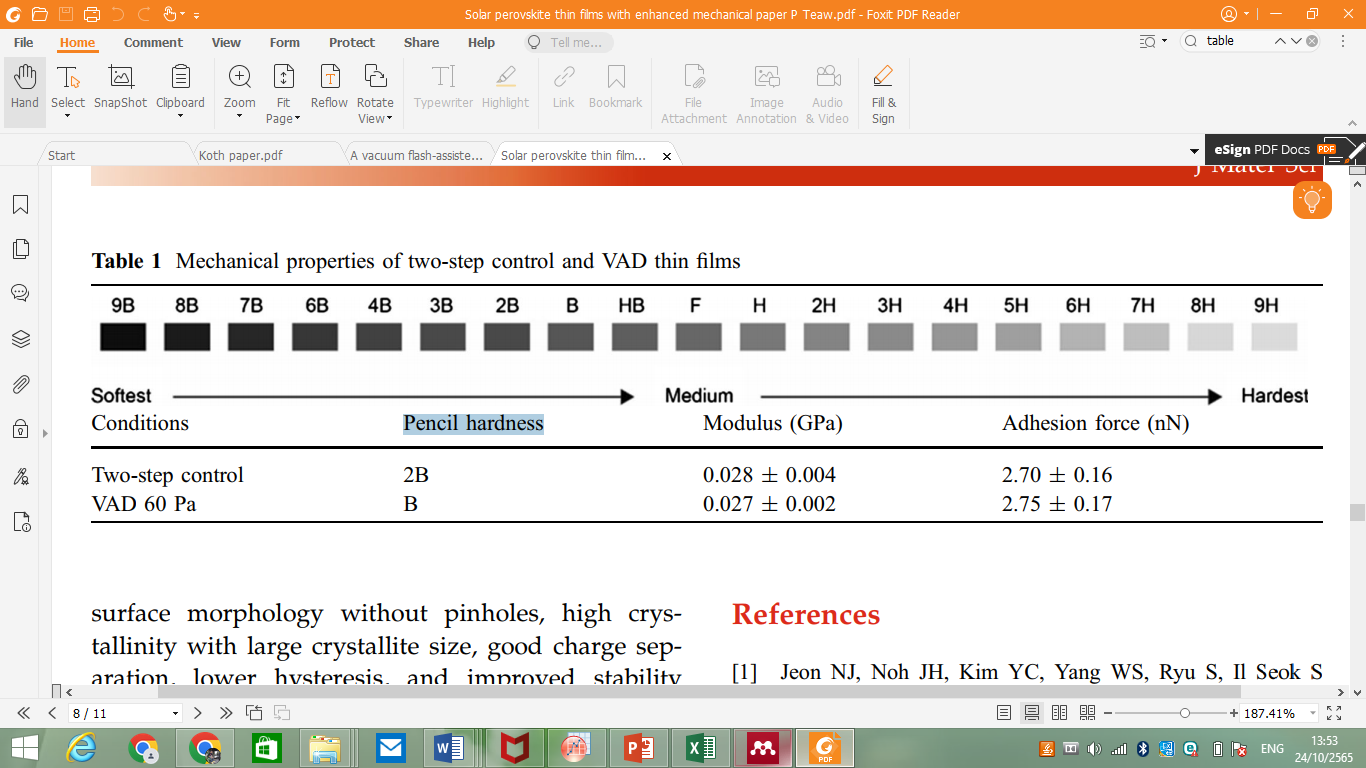**Softest-----------------------------------------------------------------------------------------------Hardest** | |
| --- | --- |
| **Condition** | **Pencil hardness** |
| Control | 2B |
| VAC | HB |
| VTA | HB |


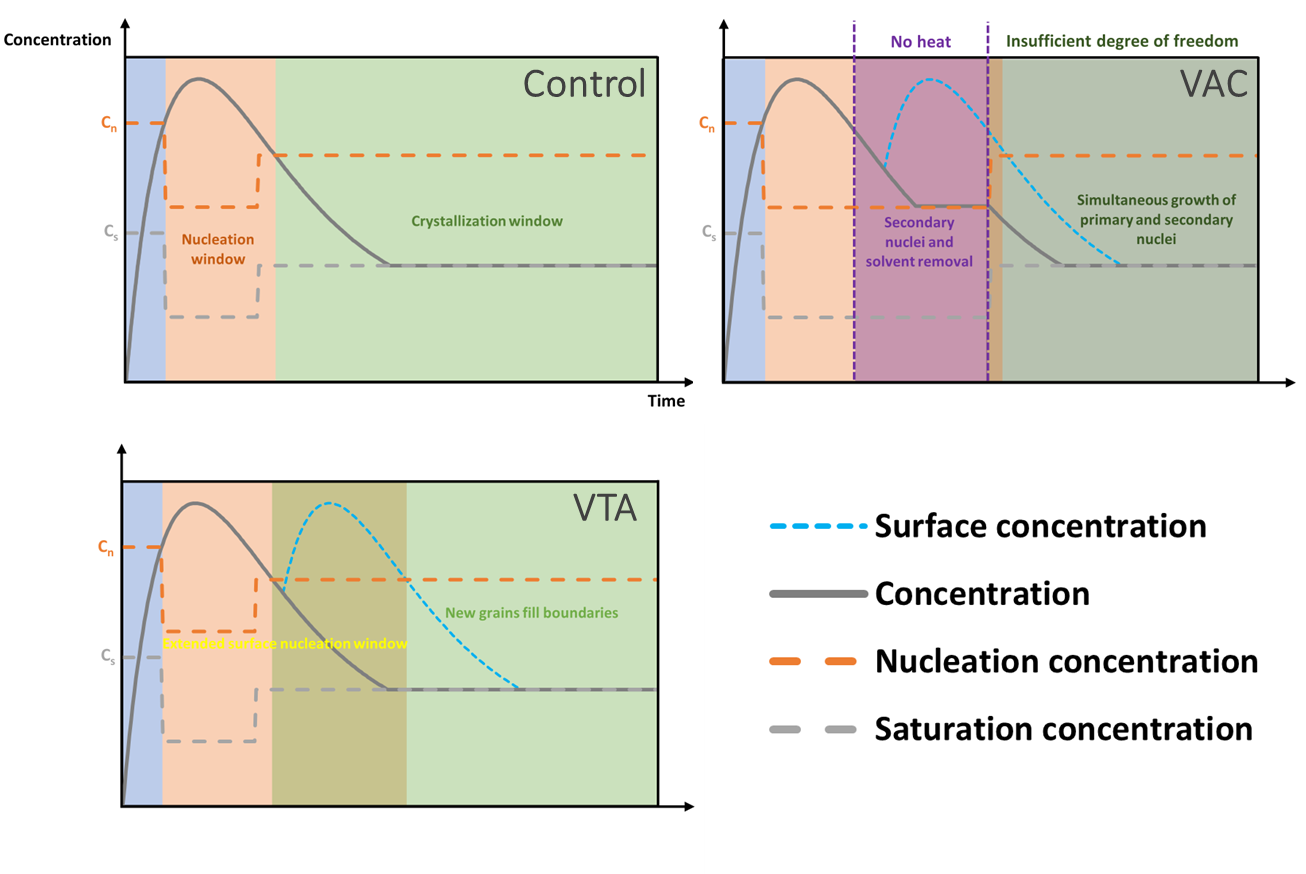


**Figure S2.** The modified LaMer model of control, VAC, and VTA. More detail about Lamer model can be found in this work [2].


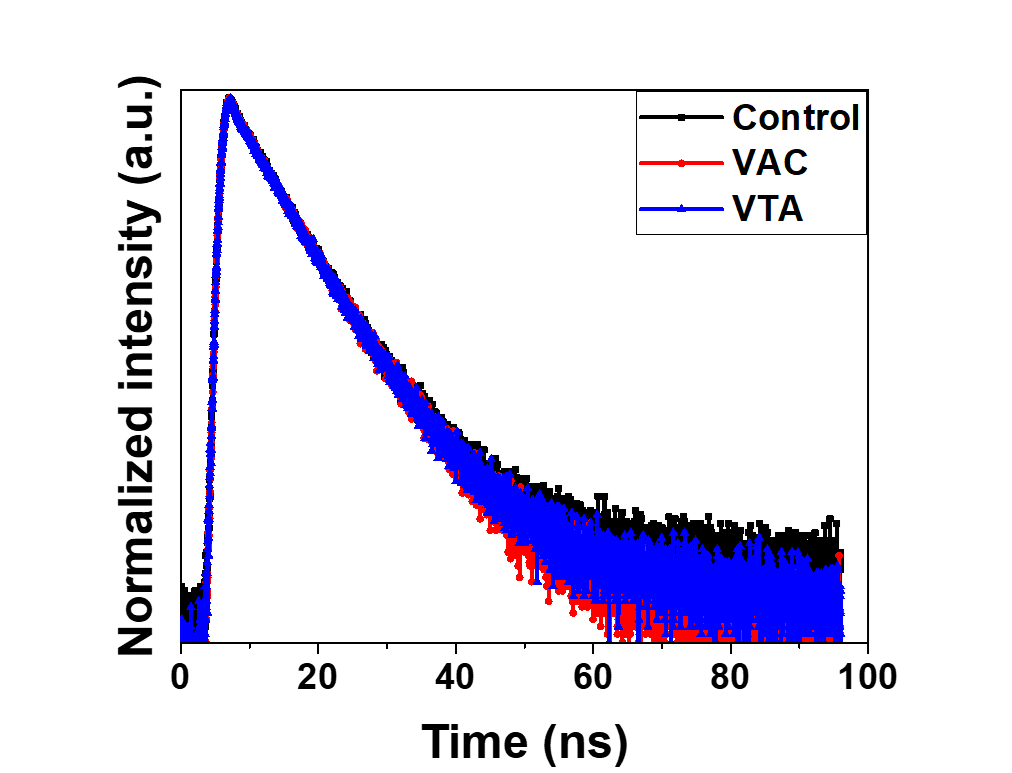


**Figure S3**. PL-lifetime results for control, VAC, and VTA samples.

**Table S2.** Photoluminescence lifetime data.

| **Condition** | $\boldsymbol{\tau}$_1_ **(ns)** | $\boldsymbol{\tau}$_2_ **(ns)** | **B_1_** | **B_2_** | $\boldsymbol{\tau}$_avg_ **(ns)** | **Chi. sq.** |
| --- | --- | --- | --- | --- | --- | --- |
| Control | 9.78 | 5.25 | 0.0066 | 0.0128 | 7.47 | 1.2360 |
| VAC | 9.98 | 5.49 | 0.0061 | 0.0132 | 7.54 | 1.2355 |
| VTA | 10.70 | 5.85 | 0.0045 | 0.0150 | 7.56 | 1.2478 |

The average lifetimes were determined by below equation [3].

$$\tau_{avg}={\sum_{i=1}^{n} \alpha_{i}\tau_{i}^{2}}/{\sum_{i=1}^{n} \alpha_{i}\tau_{i}}$$

Where$\alpha_{1}=B_{1}/(B_{1}+B_{2})$ and $\alpha_{2}=B_{2}/(B_{1}+B_{2})$.


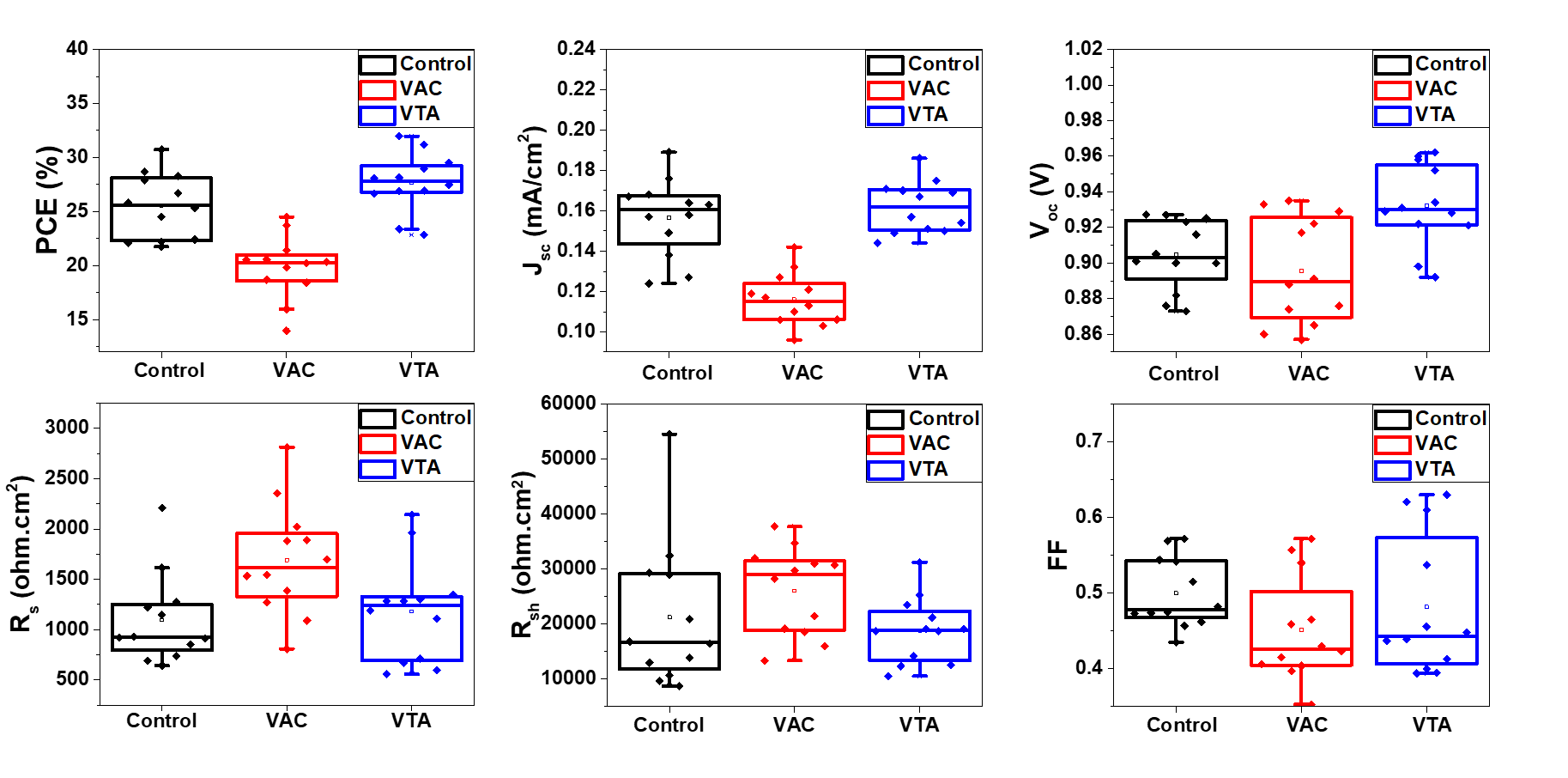
**Figure S4.** The statistics of PCE, J_sc_, V_oc_, R_s_, and R_sh_, and FF under indoor light source (1000 lux).


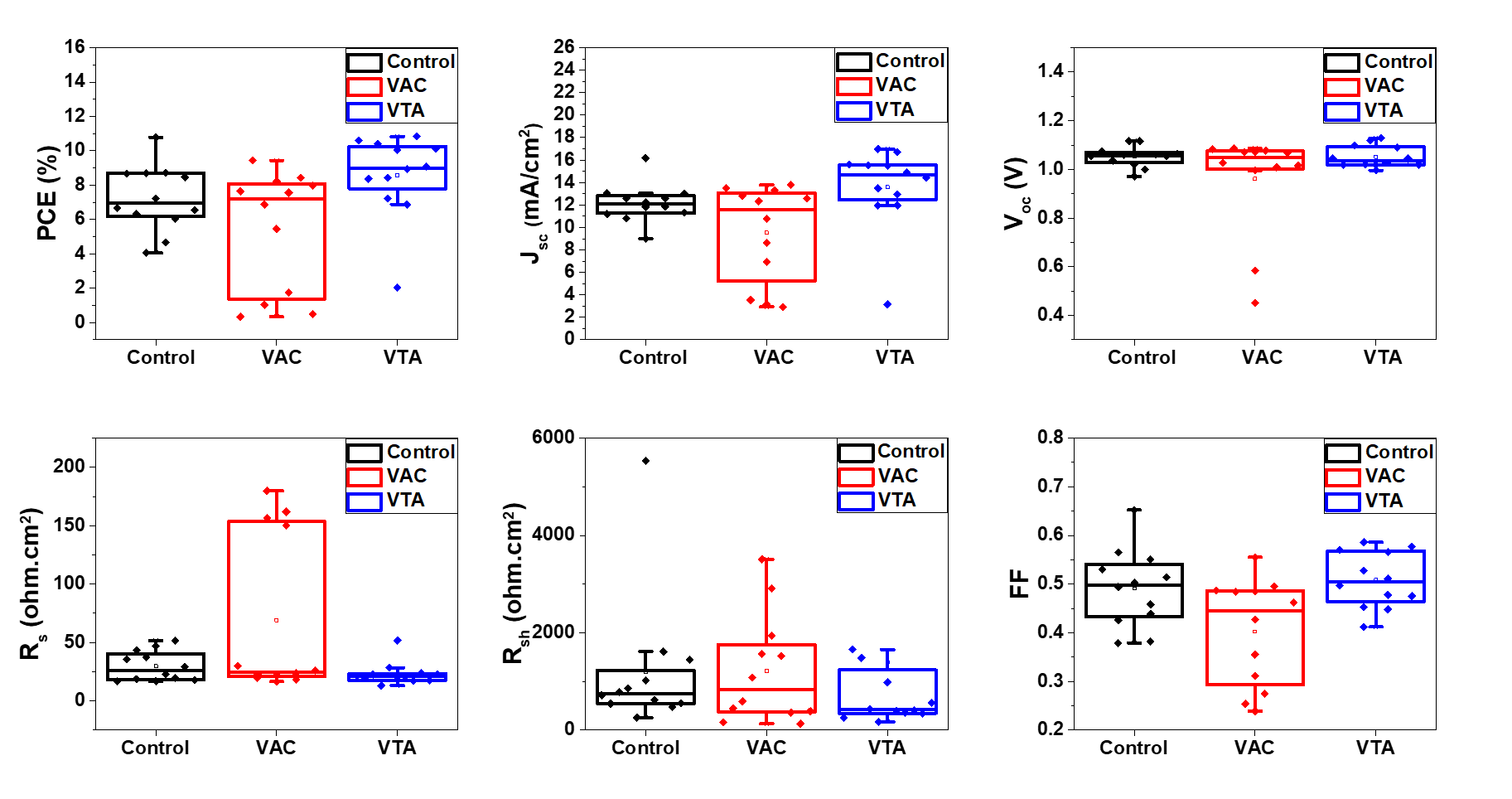


**Figure S5**. The statistics of PCE, J_sc_, V_oc_, R_s_, and R_sh_, and FF under one sun (100 mW/cm^2^).

**Table S3.** Raw data of open-circuit voltage (V_oc_), short-circuit photocurrent density (J_sc_), fill factor (FF), power conversion efficiency (PCE), R_shunt_ (R_sh_), and R_series_ (R_s_) under 1000 lux for control, VAC, and VTA.

| **Sample** | **V_oc_ (V)** | **J_sc_ (mA/cm^2^)** | **FF** | **PCE (%)** | **R_sh_ (ohm.cm^2^)** | **R_s_ (ohm.cm^2^)** |
| --- | --- | --- | --- | --- | --- | --- |
| Control | 0.87 | 0.14 | 0.46 | 22.2 | 28982 | 1615 |
| Control | 0.88 | 0.15 | 0.48 | 26.7 | 54509 | 1148 |
| Control | 0.88 | 0.12 | 0.46 | 24.5 | 32437 | 1221 |
| Control | 0.90 | 0.13 | 0.44 | 25.8 | 29339 | 2208 |
| Control | 0.92 | 0.18 | 0.54 | 28.3 | 10688 | 643 |
| Control | 0.93 | 0.17 | 0.57 | 28.7 | 12988 | 692 |
| Control | 0.93 | 0.16 | 0.57 | 27.9 | 13878 | 737 |
| Control | 0.93 | 0.19 | 0.54 | 30.7 | 20946 | 1276 |
| Control | 0.91 | 0.16 | 0.47 | 21.8 | 8729 | 929 |
| Control | 0.92 | 0.17 | 0.52 | 25.4 | 9680 | 853 |
| Control | 0.90 | 0.16 | 0.48 | 22.1 | 16791 | 918 |
| Control | 0.90 | 0.16 | 0.47 | 22.4 | 16504 | 910 |
| **Average** | **0.90** | **0.16** | **0.50** | **25.5** | **21289** | **1096** |
| VAC | 0.87 | 0.10 | 0.40 | 16.0 | 34702 | 1883 |
| VAC | 0.87 | 0.11 | 0.42 | 19.8 | 37724 | 2020 |
| VAC | 0.89 | 0.13 | 0.47 | 23.7 | 30955 | 1543 |
| VAC | 0.88 | 0.11 | 0.43 | 18.7 | 29764 | 2353 |
| VAC | 0.86 | 0.11 | 0.41 | 21.4 | 31992 | 1889 |
| VAC | 0.86 | 0.12 | 0.42 | 20.6 | 28219 | 1532 |
| VAC | 0.92 | 0.10 | 0.35 | 18.4 | 21429 | 2811 |
| VAC | 0.92 | 0.12 | 0.40 | 20.2 | 30795 | 1697 |
| VAC | 0.89 | 0.11 | 0.46 | 14.0 | 18632 | 1388 |
| VAC | 0.94 | 0.14 | 0.57 | 24.5 | 19150 | 810 |
| VAC | 0.93 | 0.13 | 0.54 | 20.5 | 16023 | 1269 |
| VAC | 0.93 | 0.12 | 0.56 | 20.4 | 13383 | 1090 |
| **Average** | **0.90** | **0.12** | **0.45** | **19.9** | **26064** | **1690** |
| **Sample** | **V_oc_ (V)** | **J_sc_ (mA/cm^2^)** | **FF** | **PCE (%)** | **R_sh_ (ohm.cm^2^)** | **R_s_ (ohm.cm^2^)** |
| VTA | 0.89 | 0.15 | 0.40 | 22.8 | 19076 | 1964 |
| VTA | 0.90 | 0.17 | 0.40 | 23.4 | 14175 | 2141 |
| VTA | 0.93 | 0.16 | 0.44 | 26.9 | 18728 | 1305 |
| VTA | 0.92 | 0.19 | 0.41 | 26.9 | 12357 | 1283 |
| VTA | 0.96 | 0.15 | 0.44 | 29.0 | 25264 | 1110 |
| VTA | 0.96 | 0.17 | 0.45 | 31.2 | 23508 | 1284 |
| VTA | 0.95 | 0.18 | 0.39 | 28.1 | 12527 | 1347 |
| VTA | 0.96 | 0.15 | 0.46 | 29.5 | 31256 | 1190 |
| VTA | 0.93 | 0.17 | 0.54 | 27.5 | 10560 | 711 |
| VTA | 0.93 | 0.15 | 0.61 | 28.1 | 21197 | 671 |
| VTA | 0.92 | 0.14 | 0.62 | 26.7 | 19128 | 599 |
| VTA | 0.93 | 0.17 | 0.63 | 32.0 | 18700 | 558 |
| **Average** | **0.93** | **0.16** | **0.48** | **27.7** | **18873** | **1180** |

**Table S4.** Raw data of V_oc_, J_sc_, FF, PCE, R_sh_, and R_s_ under AM1.5G light intensity (100 mW/cm^2^) for control, VAC, and VTA.

| **Sample** | **V_oc_ (V)** | **J_sc_ (mA/cm^2^)** | **FF** | **PCE (%)** | **R_sh_ (ohm.cm^2^)** | **R_s_ (ohm.cm^2^)** |
| --- | --- | --- | --- | --- | --- | --- |
| Control | 0.97 | 11.8 | 0.44 | 4.7 | 5538 | 47 |
| Control | 1.02 | 12.3 | 0.43 | 7.2 | 1015 | 23 |
| Control | 1.06 | 10.8 | 0.46 | 6.3 | 257 | 38 |
| Control | 1.00 | 12.6 | 0.38 | 6.0 | 619 | 29 |
| Control | 1.04 | 11.9 | 0.50 | 6.7 | 857 | 36 |
| Control | 1.06 | 16.2 | 0.55 | 10.8 | 1612 | 17 |
| Control | 1.08 | 12.6 | 0.57 | 8.7 | 782 | 19 |
| Control | 1.05 | 9.0 | 0.38 | 4.1 | 478 | 43 |
| Control | 1.05 | 11.2 | 0.65 | 8.7 | 545 | 19 |
| Control | 1.07 | 11.3 | 0.53 | 6.6 | 559 | 52 |
| Control | 1.12 | 13.1 | 0.49 | 8.5 | 720 | 17 |
| Control | 1.12 | 13.0 | 0.51 | 8.7 | 1452 | 18 |
| **Average** | **1.05** | **12.1** | **0.49** | **7.2** | **1203** | **30** |
| VAC | 1.07 | 13.3 | 0.49 | 8.2 | 2910 | 22 |
| VAC | 1.07 | 12.3 | 0.48 | 7.6 | 1942 | 23 |
| VAC | 1.08 | 13.8 | 0.56 | 9.4 | 1569 | 17 |
| VAC | 1.09 | 12.8 | 0.50 | 8.4 | 1517 | 20 |
| VAC | 1.01 | 12.6 | 0.36 | 6.9 | 3513 | 24 |
| VAC | 1.03 | 13.5 | 0.49 | 8.0 | 1087 | 18 |
| VAC | 1.07 | 8.6 | 0.43 | 5.5 | 365 | 30 |
| VAC | 1.08 | 10.8 | 0.46 | 7.6 | 593 | 26 |
| VAC | 1.02 | 6.9 | 0.28 | 1.7 | 133 | 150 |
| VAC | 1.00 | 3.1 | 0.31 | 1.0 | 451 | 162 |
| VAC | 0.59 | 3.5 | 0.24 | 0.5 | 390 | 180 |
| VAC | 0.45 | 2.9 | 0.25 | 0.3 | 157 | 156 |
| **Average** | **0.96** | **9.5** | **0.40** | **5.4** | **1219** | **69** |
| **Sample** | **V_oc_ (V)** | **J_sc_ (mA/cm^2^)** | **FF** | **PCE (%)** | **R_sh_ (ohm.cm^2^)** | **R_s_ (ohm.cm^2^)** |
| VTA | 1.02 | 12.0 | 0.57 | 8.9 | 980 | 18 |
| VTA | 1.02 | 13.5 | 0.48 | 8.4 | 170 | 21 |
| VTA | 1.00 | 16.7 | 0.51 | 6.9 | 400 | 28 |
| VTA | 1.03 | 17.0 | 0.45 | 9.1 | 438 | 24 |
| VTA | 1.13 | 15.5 | 0.45 | 10.0 | 355 | 13 |
| VTA | 1.12 | 15.5 | 0.48 | 10.4 | 1489 | 23 |
| VTA | 1.10 | 14.9 | 0.59 | 10.8 | 408 | 17 |
| VTA | 1.09 | 11.9 | 0.58 | 10.6 | 1656 | 20 |
| VTA | 1.02 | 15.6 | 0.50 | 7.2 | 341 | 23 |
| VTA | 1.04 | 14.4 | 0.41 | 8.4 | 254 | 21 |
| VTA | 1.05 | 3.1 | 0.57 | 10.1 | 558 | 17 |
| VTA | 1.02 | 13.6 | 0.53 | 2.0 | 9720 | 52 |
| **Average** | **1.05** | **13.0** | **0.51** | **8.6** | **1397** | **23** |

**
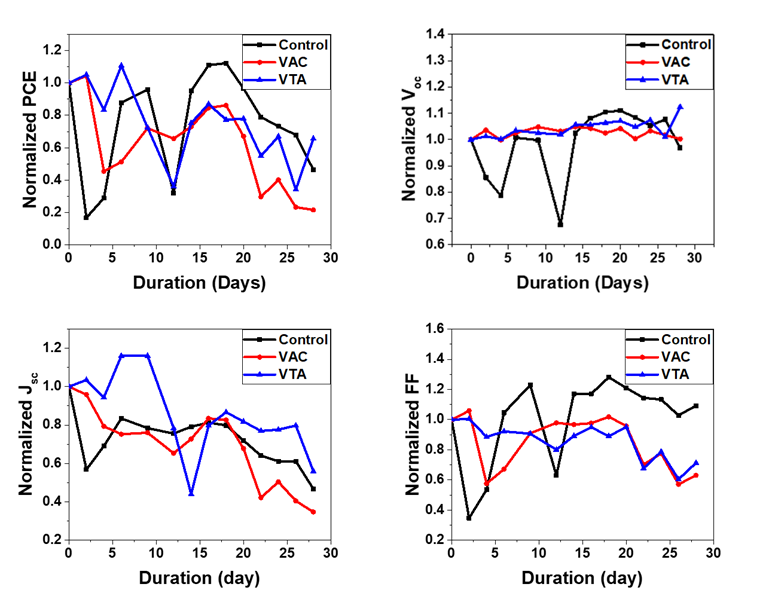
**

**
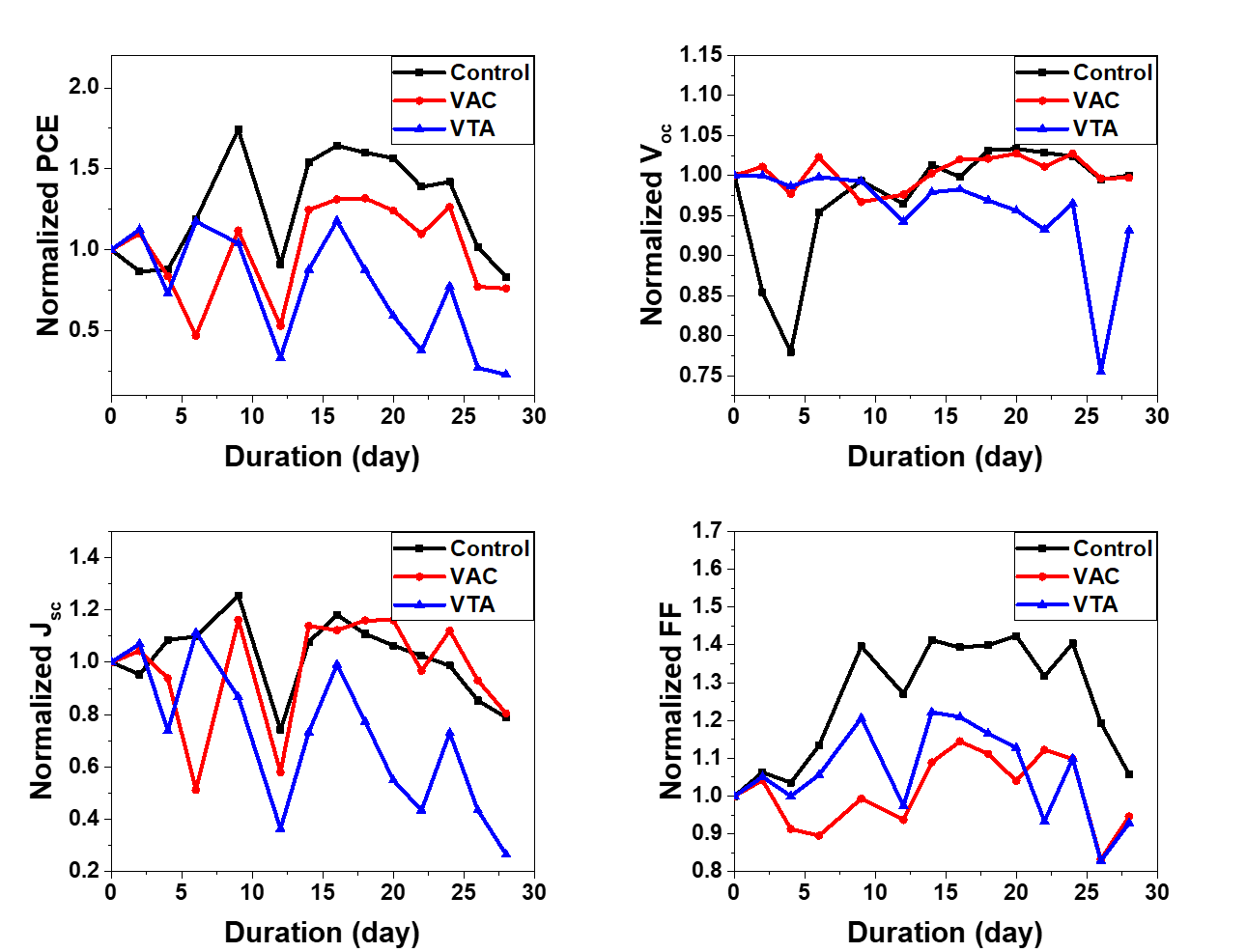
Figure S6.** The normalized stability of PCE, V_oc_, J_sc_, and FF under indoor light source (1000 lux).

**Figure S7.** The normalized stability of PCE, V_oc_, J_sc_, and FF under one sun source (100 mW/cm^2^).

**
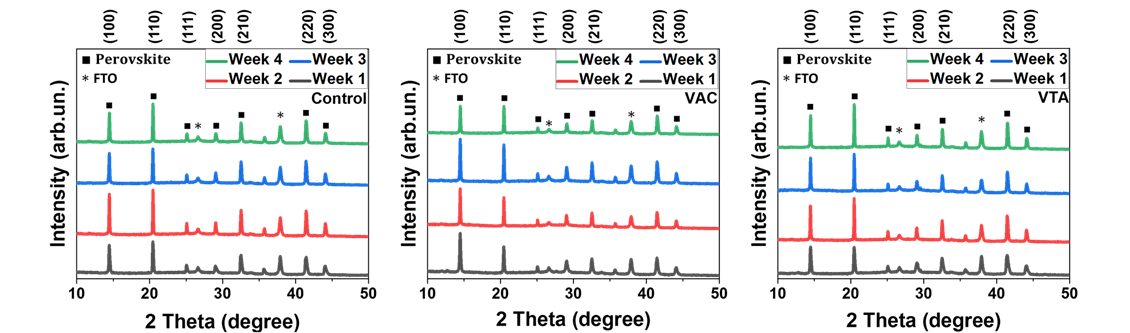
**

**Figure S8.** XRD patterns of only-film stability of control, VAC, and VTA samples.

Furthermore, we investigated long-term device stability of the highest performing devices as shown in Fig. S6 and S7. The devices with encapsulation were kept under 1000 lux for 8 hours/day and measured under ambient air under both 1000 lux and one sun. The device parameters were tested every 2 days by measuring J-V curves. Unfortunately, we do not observe any improvement from the VTA process possibly due to other important factors influencing device stability like the use of spiro-OMeTAD, encapsulation materials, and adhesion of carbon electrode. To understand the film-only stability, fresh, 1-week, 2-week, 3-week, and 4-week aged perovskite films under 30% RH condition were characterized with XRD; similar features without any PbI_2_ formation were observed for all conditions. These XRD results confirm similar materials stability for control, VAC, and VTA.

**References**

[1] Pinsuwan, K. *et al.* Solar perovskite thin films with enhanced mechanical, thermal, UV, and moisture stability via vacuum-assisted deposition. *J. Mater. Sci.* **55**(8), 3484–3494. https://doi.org/10.1007/s10853-019-04199-9 (2020).

[2] Yi, Z. et al. Will organic-inorganic hybrid halide lead perovskites be eliminated from optoelectronic applications? Nanoscale Adv.

**1**(4), 1276–1289. https://doi.org/10.1039/c8na00416a (2019).

[3] Zhang, M. *et al.* Composition-dependent photoluminescence intensity and prolonged recombination lifetime of perovskite CH_3_NH_3_PbBr_3-x_Cl_x_ films. *Chem. Commun.* **50**(79),11727–11730. https://doi.org/10.1039/c4cc04973j (2014).
